# Immunity and mental illness: findings from a Danish population-based immunogenetic study of seven psychiatric and neurodevelopmental disorders

**DOI:** 10.1038/s41431-019-0402-9

**Published:** 2019-04-11

**Authors:** Ron Nudel, Michael E. Benros, Morten Dybdahl Krebs, Rosa Lundbye Allesøe, Camilla Koldbæk Lemvigh, Jonas Bybjerg-Grauholm, Anders D. Børglum, Mark J. Daly, Merete Nordentoft, Ole Mors, David M. Hougaard, Preben Bo Mortensen, Alfonso Buil, Thomas Werge, Simon Rasmussen, Wesley K. Thompson

**Affiliations:** 1 Institute of Biological Psychiatry, Mental Health Centre Sct. Hans, Mental Health Services Copenhagen, Roskilde, Denmark; 2 iPSYCH, The Lundbeck Foundation Initiative for Integrative Psychiatric Research, Copenhagen, Denmark; 3 Mental Health Centre Copenhagen, University of Copenhagen Hospital, Copenhagen, Denmark; 4 Department of Bio and Health Informatics, Technical University of Denmark, Kongens Lyngby, Denmark; 5 Center for Neonatal Screening, Department for Congenital Disorders, Statens Serum Institut, Copenhagen, Denmark; 6 Department of Biomedicine, Aarhus University and Centre for Integrative Sequencing, iSEQ, Aarhus, Denmark; 7 Aarhus Genome Center, Aarhus, Denmark; 8 Stanley Center for Psychiatric Research, Broad Institute of Harvard and MIT, Cambridge, MA USA; 9 Department of Clinical Medicine, Faculty of Health and Medical Sciences, University of Copenhagen, Copenhagen, Denmark; 10 Psychosis Research Unit, Aarhus University Hospital, Risskov, Denmark; 11 National Center for Register-Based Research, Aarhus University, Aarhus, Denmark; 12 Novo Nordisk Foundation Center for Protein Research, Faculty of Health and Medical Sciences, University of Copenhagen, Copenhagen, Denmark; 13 Department of Family Medicine and Public Health, Division of Biostatistics, University of California, San Diego, CA USA

**Keywords:** Psychiatric disorders, Immunogenetics

## Abstract

Human leukocyte antigen (HLA) genes encode proteins with important roles in the regulation of the immune system. Many studies have also implicated HLA genes in psychiatric and neurodevelopmental disorders. However, these studies usually focus on one disorder and/or on one HLA candidate gene, often with small samples. Here, we access a large dataset of 65,534 genotyped individuals consisting of controls (*N* = 19,645) and cases having one or more of autism spectrum disorder (*N* = 12,331), attention deficit hyperactivity disorder (*N* = 14,397), schizophrenia (*N* = 2401), bipolar disorder (*N* = 1391), depression (*N* = 18,511), anorexia (*N* = 2551) or intellectual disability (*N* = 3175). We imputed participants’ HLA alleles to investigate the involvement of HLA genes in these disorders using regression models. We found a pronounced protective effect of DPB1*1501 on susceptibility to autism (*p* = 0.0094, OR = 0.72) and intellectual disability (*p* = 0.00099, OR = 0.41), with an increased protective effect on a comorbid diagnosis of both disorders (*p* = 0.003, OR = 0.29). We also identified a risk allele for intellectual disability, B*5701 (*p* = 0.00016, OR = 1.33). Associations with both alleles survived FDR correction and a permutation procedure. We did not find significant evidence for replication of previously-reported associations for autism or schizophrenia. Our results support an implication of HLA genes in autism and intellectual disability, which requires replication by other studies. Our study also highlights the importance of large sample sizes in HLA association studies.

## Introduction

Proteins of the major histocompatibility complex (MHC) in humans are encoded by the human leukocyte antigen (HLA) region on chromosome 6. This region is highly dense in genes and highly polymorphic, and many genetic variants lying within it have been implicated in human diseases, such as autoimmune disorders and infections [1]. Proteins encoded by HLA genes have important roles in immune reaction and are divided into three classes. In this study, we focus on genes from HLA classes I and II. HLA class I genes encode proteins that present endogenous antigens (produced inside the cell), and HLA class II genes encode proteins that present antigens from outside the cell to T cells [2]. Although less clear than the connection between autoimmune diseases and the immune system, the connection between psychiatric and neurodevelopmental disorders and the immune system is supported by increasing evidence in past decades, from associations of autoimmune diseases and allergies with schizophrenia and autism spectrum disorder (ASD) to dysregulation of immune-related genes or cytokine levels in those disorders [3]. While many robust associations of HLA genes with autoimmune diseases have been reported [4], several class I and class II HLA genes have also been implicated in psychiatric and neurodevelopmental disorders, albeit with uneven reproducibility. Specifically, the A2 allele of *HLA-A*, several *HLA-B* alleles, as well as *HLA-DRB1* DR4 have been reported to be associated with ASD [5–8], the latter also being associated with attention deficit hyperactivity disorder (ADHD) [9]. For schizophrenia, associations with many HLA genes have been reported. In an extensive review [10], Wright et al. propose that much of the evidence for these associations is weak due to problems in study design. More recently, a study reported variants in the *C4* loci (part of the complement system) to be associated with schizophrenia [11]. The involvement of HLA genes in either affective disorders or anorexia has not been robustly established, with studies mostly originating from the 1980s and reaching different conclusions [12–16]. In this study, we test for association between seven major psychiatric or neurodevelopmental disorders and seven HLA genes in an unbiased approach, using a genetically homogeneous sample from the Danish Integrative Psychiatric Research (iPSYCH) Consortium. Additionally, we attempt to replicate previously reported associations.

## Methods

### Participants, SNP arrays, and quality control procedures

The individuals included in this study are part of the iPSYCH consortium’s sample of Danish individuals selected for having a psychiatric diagnosis or as random population controls [17]. Quality control (QC) procedures were followed in order to remove individuals of divergent ancestry and to keep only unrelated cases and controls. This was achieved by using Danish registry data on family history and through a genetic principal component analysis (PCA). Before QC, our sample included 78,050 subjects genotyped in 23 of the original 25 waves used in subsequent QC and downstream analyses. The QC procedures leading to the sub-sample analyzed here were originally performed for another study and are described extensively elsewhere [18]. In short, PCAs were performed with the iPSYCH individuals and several 1000 Genomes samples as a reference panel: in the first step, both datasets were used to create a PC space, whereupon individuals whose parents and grandparents were known to have been born in Denmark (based on Danish birth records) were used as a reference for removing individuals deviating from the multivariate mean of the joint distribution of the first ten principal components (PCs). This was then repeated using only the remaining iPSYCH individuals to account for subtle within-population differences. Individuals were also removed based on missingness (1%) and abnormal heterozygosity or ambiguous sex, based on genetic markers. Individuals who were identified as duplicates were also removed. Lastly, individuals found to be related to others (first and second degrees) were removed, whereby cases and then individuals with a higher genotype call rate were prioritized. Following this, a new PCA was performed and the new PCs were used as covariates in downstream analyses. The total number of individuals in the post-QC sample in this study was 65,534. Since this study did not use the SNP data directly, we discuss them only briefly here. Samples were genotyped on the Illumina Infinium PsychArray v1.0. The QC procedure employed consisted of the following thresholds: SNP missingness of 5% (before sample removal); subject missingness of 2%; autosomal heterozygosity deviation | Fhet | of 0.2; SNP missingness of 2% (after sample removal); and SNP Hardy-Weinberg equilibrium *p*-value of 10^−6^.

The phenotype groups included in this study were as follows: controls (no psychiatric or neurodevelopmental diagnoses, as per ICD-10 codes F00-F99); schizophrenia cases (F20); bipolar disorder (BPD) cases (F30-F31); single and recurrent depressive disorder cases (F32-F33); anorexia nervosa cases (F50.0); ASD cases (F84.0, F84.1, F84.5, F84.8, and F84.9), ADHD cases (F90.0) and intellectual disability (ID) cases (F70-F79). The first six case categories were the primary phenotype groups in the iPSYCH case sample, together with a random population sample from which the controls were selected. A diagnosis of ID is a secondary phenotype in this sense. Diagnoses are based on records dating up to 2013 from the Danish Psychiatric Central Research Register. While controls have no psychiatric diagnosis in the register, some cases are diagnosed with more than one disorder, and we discuss one example of this relevant to our results. Otherwise, we did not exclude individuals with comorbid diagnoses. Sample sizes are given in Table 1. For subtypes of ID and for anorexia only, diagnoses from the Danish National Patient Register that were not present in the psychiatric register were available for a small number of individuals (30 for ID and 83 for anorexia). For significant HLA associations identified in this study, we repeat the analyses after excluding the relevant group of discordant individuals.

**Table 1:** Sample sizes across controls and case groups

Group	Sample count
Controls	19,645
ASD	12,331
ADHD	14,397
Schizophrenia	2401
BPD	1391
Single and recurrent depression	18,511
Anorexia	2551
ID	3175
ASD excluding ID	10,579
ID excluding ASD	1423
Both ASD and ID	1752

### Generation and processing of exome data

A subset of the iPSYCH sample was exome-sequenced. Sequencing libraries were produced using a custom adaptation of the Illumina Rapid Target Kit (Illumina ICE Broad Exome) and sequenced on Illumina Hiseqs. The raw reads were mapped using bwa aln (v5.9) with the parameters -q 5 -l 32 -k 2 to GRCh37 including unplaced and unlocalized contigs and Epstein-barr (NC_007605.1). Thereafter PCR duplicates were removed using picard MarkDuplicates, combined per sample and realigned across indels using GATK IndelRealigner [19].

### Imputation of HLA alleles

SNP data were used to impute HLA types with a four-digit resolution for: *HLA-A, HLA-B, HLA-C, HLA-DRB1, HLA-DQA1, HLA-DQB1*, and *HLA-DPB1*. The HLA imputation was performed with HIBAG v1.3 [20] using a pre-trained four-digit European ancestry model based on the PsychArray-B genotype platform (downloaded from: http://zhengxwen.github.io/HIBAG/hibag_index.html). The alleles were imputed based on 385–556 sites, depending on the locus, and each wave was imputed separately. Waves were thereafter combined. Additionally, we called class I alleles from the exome-sequencing data of 500 randomly sampled individuals using Polysolver [21]. When running Polysolver, a reference dataset of Caucasian individuals was used, without frequencies. Kolmogorov-Smirnov tests were performed in R [22] comparing posterior probability distributions from HIBAG for alleles highlighted in the statistical analyses as described below against all other alleles for the particular locus. The posterior probability threshold for HLA alleles used in downstream analyses was 0.9.

The iPSYCH data are stored in a national HPC facility in Denmark. The iPSYCH initiative is committed to providing access to these data to the scientific community, in accordance with Danish law. Researchers may be granted access upon request to the iPSYCH management.

### Statistical analysis

Allele counts for each HLA subtype are used as variables in two types of analyses: a gene-based test and an allele-specific test, with covariates for: age, age squared (to account for non-linearity with age), sex, and the first ten PCs from the PCA, to account for differences in genetic ancestry. In both analyses, HLA alleles are coded as allele counts (0, 1 or 2). The iPSYCH samples in the final dataset were genotyped in 23 waves. In order to determine whether the genotyping waves had any effect on association, we performed regressions of the wave category on the HLA alleles (23 regressions in total, where in each one a given wave is tested against all other waves). As the waves were selected by year of birth, age (and psychiatric diagnosis) could be confounded with them. To test for any independent effect of the waves, we performed the regressions in the psychiatric control subset with a covariate for age. The results were subject to FDR correction. None of the HLA subtypes showed significant association with any wave (minimum *q* = 0.394). We therefore do not use a covariate for wave, but for the top associations we do examine how adding this covariate affects the result. In all (non-stratified) analyses, groups with a psychiatric or neurodevelopmental diagnosis are compared against controls (with no psychiatric or neurodevelopmental diagnosis). For ID, we also perform “stratified” analyses for each of the other diagnoses (note that these are not mutually-exclusive subsets of the original sample). All statistical analyses are implemented in R, and the regressions were performed with the glm function.

### Gene-based tests

The gene-based tests serve as omnibus tests and assess whether the inclusion of all alleles (as separate variables) in the model improves the model significantly. We do not draw conclusions on specific alleles from those tests, but, rather, we use them as a starting point for potential post hoc tests. They consist of logistic regressions including all alleles (as separate variables, i.e., each individual has a count of 0, 1 or 2 alleles per each imputed allele passing QC) of a given HLA gene plus the covariates (full model) or the covariates alone (null model). The two models are compared using a likelihood ratio test (LRT). Reported *p*-values are from the LRT chi-squared statistics. These analyses thus determine the overall association of a given HLA gene with the disorder of interest by assessing whether the inclusion of the allele variables significantly explains more of the variance in the outcome. To account for multiple testing, we employ a Bonferroni correction but also consider false discovery rate (FDR) *q*-values, as implemented in QVALUE v1.0 [23]. All *p*-values from the gene-based tests across all disorders were used in the generation of *q*-values using the bootstrap method.

### Allele-specific tests

**Fig. 1:**
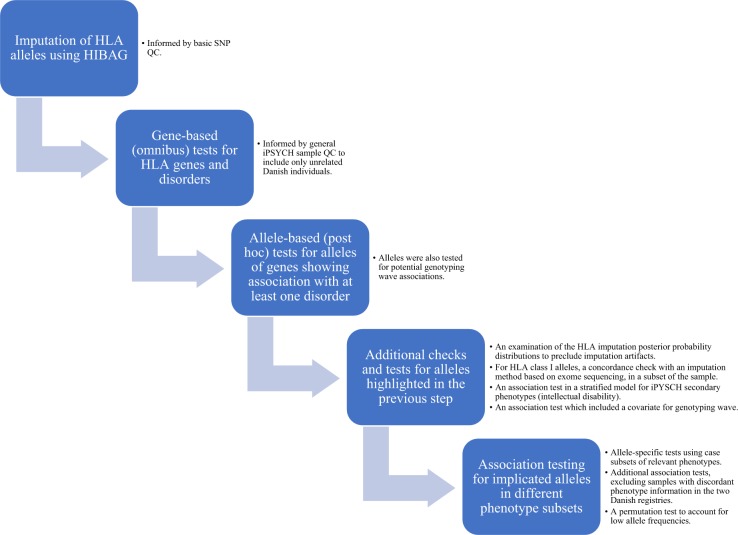
An outline of the study design and performed analyses. See Methods section for more detailed information

We perform post hoc allele-specific tests for genes that show a significant association with at least one disorder, where each allele of the gene is tested separately in a logistic regression with the same covariates (and the relevant disorder as the outcome). Reported *p*-values pertain to whether the coefficient (log-odds ratio) for the allele is different from zero (Wald test). For these tests we report the odds ratio (OR) and 95% confidence interval (CI). We also use this approach to test for association with alleles previously reported to be implicated in the investigated disorders. In addition to a Bonferroni correction, we compute *q*-values based on the distributions of *p*-values for all successfully tested alleles of each gene, both per HLA gene-disorder association and across all groups. This applies to genes implicated in the gene-based analyses as well as genes implicated in previous studies, in the replication analyses. Where possible, the bootstrap method is used; this depended on the number of *p*-values and their distribution. Otherwise, we set the tuning parameter *λ* = 0, resulting in a conservative analysis. Lastly, for alleles highlighted in the above analyses, we perform permutation tests using glmperm [24] in R with 10,000 permutations. Figure 1 includes an outline of the study design and performed analyses.

## Results

### Imputation of HLA alleles in the Danish cohort

**Fig. 2:**
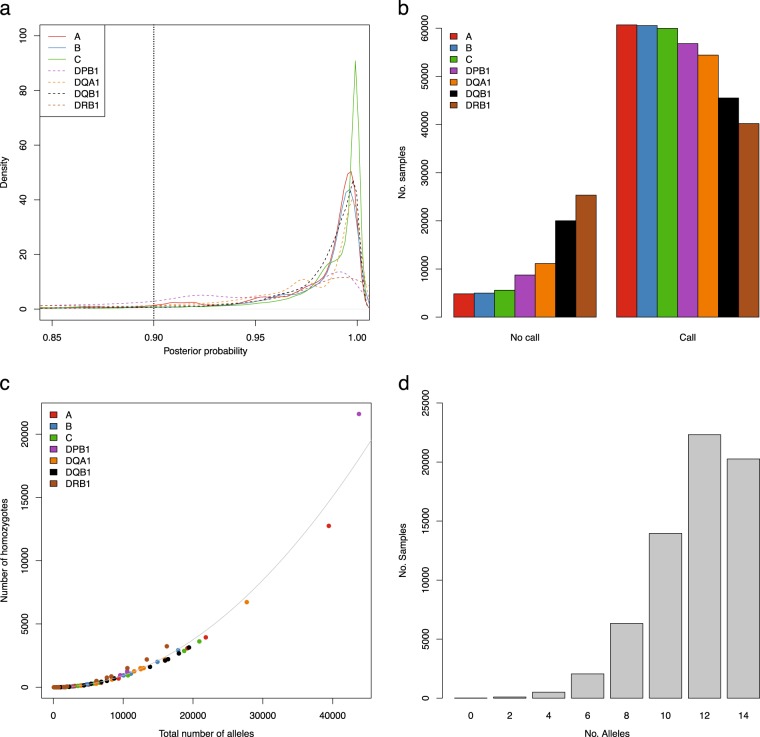
Imputation of HLA alleles. **a** Posterior probability densities per HLA locus from HIBAG imputation. HLA class I are solid lines, HLA class II loci are represented as dotted lines, vertical dotted line at 0.90 represents filtering threshold. Color coding: A: red, B: blue, C: green, DPB1: magenta, DQA1: orange, DQB1: black and DRB1: brown. **b** Number of samples that were removed (no call) or kept (call) for the seven HLA loci, color coding as in (**a**). **c** Correlation between the total number alleles called and the number of homozygous individuals for a given allele. Each allele is color coded according the locus, color as in (**a**). **d** Number of alleles called per sample after filtering for posterior probability. The maximum number of alleles that can be called per sample across all seven loci is 14

For the three HLA class I loci (A, B, and C) and four HLA class II loci (DPB1, DQA1, DQB1, and DRB1) iPSYCH genotype chip data were used to impute HLA alleles with HIBAG. We imputed two alleles at each locus resulting in diploid allele calls for each of the seven HLA genes and filtered for a posterior probability threshold of 0.9 (Fig. 2a). This approach removed between 4852 and 25,331 individuals for specific HLA loci, with *HLA-A* having the largest number of high-confidence imputations, and *HLA-DRB1* having the lowest (Fig. 2b). In general, HIBAG was able to call alleles with high posterior probabilities, but for some alleles low posterior probabilities were obtained (Supplementary Figs. S1–S2), and the latter were more likely to be removed. To validate the imputations, we used PolySolver to call class I alleles for 500 randomly-sampled individuals for whom we have exome-sequencing data. Here we found a concordance rate of 0.981, 0.986, and 1.00 for *HLA-A*, *HLA-B*, and *HLA-C*, respectively, showing a very good overlap between the two methods and datasets. Furthermore, we investigated the rate of homozygous calls and found, as expected, a quadratic correlation between the total number of alleles called and the number of individuals homozygous for a particular allele (*R*^2^-adjusted: 0.99, *p* < 10^−16^) (Fig. 2c). In the final set of imputed HLA-alleles, 56,535 individuals (86.3%) had alleles that had passed QC for at least 5 loci (Fig. 2d). In total, the following numbers of alleles were imputed for HLA-A, HLA-B, HLA-C, HLA-DPB1, HLA-DQA1, HLA-DQB1, and HLA-DRB1, respectively: 31, 65, 30, 21, 15, 17, and 43. After allele and sample QC, the following numbers of alleles remained: 24, 42, 21, 15, 12, 14, and 27. The distributions of HLA allele counts and frequencies in case and control groups can be found in Supplementary Table S1. Supplementary Table S2 contains the amino acid sequence alignments for exons 2–3 (class I genes) or exon 2 (class II genes), the antigen binding domains, which correspond to the HLA allele resolution in this study, for all imputed alleles passing the allele QC, as well as the genomic sequence of all individual HLA alleles at a full resolution from http://hla.alleles.org/alleles/text_index.html (accessed on 6 March 2019) [25, 26], for reference purposes.

### Associations of HLA alleles with neurodevelopmental or psychiatric disorders

Gene-based LRT chi-squared *p*-values for all disorders are given in Table 2. After a strict Bonferroni correction for 49 tests, only the association between *HLA-DPB1* and ID survived. FDR correction, however, results in three significant tests: ID with *HLA-B* (*q* = 0.047) and *HLA-DPB1* (*q* = 0.004), and ASD with *HLA-DPB1* (*q* = 0.047). Our post hoc analyses thus investigated which alleles in *HLA-B* and *HLA-DPB1* might be individually significantly associated with ID or ID and ASD, respectively. In the framework of the Bonferroni correction, that is, if only the association between *HLA-DPB1* and ID is considered, one allele, DPB1*1501 ID (*p* = 0.00099, OR = 0.41, 95% CI: 0.23, 0.67), remains significant after correction for the number of *HLA-DPB1* alleles tested (*N* = 15). In the framework of FDR-significant associations, for *HLA-B* we found one allele, B*5701, to be significantly associated with increased risk of ID (*p* = 0.00016, *q* = 0.004, OR = 1.33, 95% CI: 1.14, 1.53). DPB1*1501 was significantly associated with reduced risk in ID (*q* = 0.015). The same allele showed a near-FDR-significant association in the same direction in ASD (*p* = 0.0094, *q* = 0.07, OR = 0.72, 95% CI: 0.56, 0.92). The above *q*-values were computed per gene-disorder association due to the large difference in allelic variation across genes; when *p*-values across all gene-disorder groups are considered simultaneously, the lowest *q*-value was 0.13, obtained for the above three allele-disorder associations and six others, suggesting that 1–2 of them are likely to be false positives.

**Table 2:** Association *p*-values for the gene-based tests across all disorders and HLA genes

Disorder	*HLA-A*	*HLA-B*	*HLA-C*	*HLA-DRB1*	*HLA-DQA1*	*HLA-DQB1*	*HLA-DPB1*
ADHD	0.9473	0.4631	0.7223	0.7758	0.694	0.7355	0.1623
Anorexia	0.8448^b^	0.1232^b^	0.9765	0.8317	0.6557	0.2731	0.286
ASD	0.3388	0.708	0.613	0.2499	0.1922	0.5358	0.00253^a^
BPD	0.5202	0.9946^b^	0.1097	0.2729^b^	0.3441	0.2113	0.5345
ID	0.1143	0.003425^a^	0.3553	0.6607^b^	0.7052	0.182	0.0001035^a^
Schizophrenia	0.1392	0.2337	0.8546	0.6014	0.834	0.4088	0.177
Single and recurrent depression	0.9107	0.5133	0.4299	0.3706	0.2282	0.04346	0.2829

^a^Tests that obtained *q* ≤ 0.05

^b^Tests where, in the full model, the algorithm did not converge or extreme probabilities were obtained

To ensure that these associations were not spurious artifacts of the imputation process, we investigated the posterior probability distributions from HIBAG for these two alleles. We found their posterior probability distribution to have different trends compared to all other alleles from the same loci (Kolmogorov-Smirnov test: *p* < 10^−16^). Examining the direction of the trend, we found no evidence of their being worse (*p* < 10^−16^), but there was evidence of their being better (*p* = 1 and *p* = 0.11), corresponding well to the actual distributions (Supplementary Fig. S3). This can be seen in the figure, where, in both cases, the red line, corresponding to the allele in question, shows a distribution that is right-shifted compared to all the other alleles of the same gene (blue line), representing a higher density towards posterior probabilities closer to 1. The number of alleles that these two were compared to were 54 alleles for B*5701 and 20 for DPB1*1501 (all alleles passing the allele QC for the two genes). Additionally, we investigated the concordance for B*5701 specifically and found a concordance rate of 1 between HIBAG and PolySolver for this allele (39 alleles called in 500 individuals). We are not able to determine concordance for DPB1*1501, as PolySolver does not call HLA class II genes. The *p*-values of the allele-specific tests for all tested alleles in these gene-disorder groupings can be found in Supplementary Table S3. Since ID was not one of the primary phenotypes for which iPSYCH cases were selected, there could be some ascertainment bias with ID in the sense that more individuals with ID in the sample are also expected to have another iPSYCH diagnosis than if they had been selected primarily for ID. We therefore tested both DPB1*1501 and B*5701 for association with ID only within iPSYCH cases for each of the other six disorders (separately). For DPB1*1501, the only nominally significant association (*p* = 0.01) was within ASD cases. For B*5701, there were two nominally significant associations, with ADHD (*p* = 0.01) and depression (*p* = 0.049). In all cases the observed association had the same trend with ID as in the non-stratified analyses. Similarly, adding a covariate for wave for the associations of DPB1*1501 and B*5701 with ID did not alter the results significantly, with ORs of 0.39 and 1.32, and *p*-values of 0.00052 and 0.00021, respectively.

While the DPB1*1501 association with ASD is only nominally significant, given the phenotypic overlap between ID and ASD, it is interesting that the same allele is at least nominally associated with both disorders in the same direction. We therefore investigated this association further by repeating both types of tests using the subset of individuals with ASD but without ID, the subset of individuals with ID but without ASD, and the subset of individuals with both diagnoses, labeled ASD^+^ID^−^, ASD^−^ID^+^, and ASD^+^ID^+^, respectively. Note, while these analyses allowed us to investigate which condition drove the observed associations, they also resulted in reduced samples sizes (Table 1). The gene-based tests for *HLA-DPB1* with ASD^+^ID^−^, ASD^−^ID^+^, and ASD^+^ID^+^ remained at least nominally significant, with p-values of 0.0046, 0.028, and 0.0012, respectively. The gene-based test for *HLA-B* with ASD^−^ID^+^ obtained a *p*-value of 0.0036, and the test with ASD^+^ID^+^ obtained a *p*-value of 0.059. The allele-specific analyses showed that DPB1*1501 was not significantly associated with ASD^+^ID^−^ (*p* = 0.058, OR = 0.78, 95% CI: 0.61, 1.01) or with ASD^−^ID^+^ (*p* = 0.09, OR = 0.56, 95% CI: 0.27, 1.03), but it was significantly associated with ASD^+^ID^+^ (*p* = 0.003) and in the same direction as before (OR = 0.29, 95% CI: 0.11, 0.6). In contrast, B*5701 showed an even stronger association than before with increased risk of ASD^−^ID^+^ (*p* = 1.37 × 10^−5^, OR = 1.54, 95% CI: 1.26, 1.86), despite the fact that the subset of individuals with a diagnosis of ID but without a diagnosis of ASD was the smallest of the three in terms of sample size (Table 1). It was not significantly associated with ASD^+^ID^+^ in the allele-specific test (*p* = 0.167, OR = 1.15, 95% CI: 0.94, 1.41). A summary of all follow-up allele-specific associations that were at least nominally significant (*p* ≤ 0.05) can be found in Table 3. Considering these tests, the associations between DPB1*1501 and ASD^+^ID^+^ and B*5701 and ASD^−^ID^+^ survive Bonferroni correction for 10 tests. None of the associations in Table 3 were much affected by the exclusion of the 30 register-discordant ID cases (in fact they all obtained slightly more significant *p*-values). Given the low allele frequencies particularly of DPB1*1501 but also of B*5701, we performed permutation tests, which could help address this issue. We obtained significant permutation *p*-values for DPB1*1501 with ID and ASD^+^ID^+^ (*p* = 2 × 10^−4^ and 4 × 10^−4^, respectively), and for B*5701 with ID and ASD^−^ID^+^ (*p* = 2 × 10^−4^ in both cases).

**Table 3:** Nominally-significant associations (*p* ≤ 0.05) from the follow-up allele-specific tests

Disorder	Gene	Allele	*p*-value	OR	95% CI for OR
ID	HLA-B	B*5701	0.00016	1.33	1.14, 1.53
ID	HLA-DPB1	DPB1*1501	0.00099	0.41	0.23, 0.67
ASD	HLA-DPB1	DPB1*1501	0.0094	0.72	0.56, 0.92
ASD^+^ID^+^	HLA-DPB1	DPB1*1501	0.003	0.29	0.11, 0.6
ASD^−^ID^+^	HLA-B	B*5701	1.37 × 10^−5^	1.54	1.26, 1.86

### Replication of previous HLA associations with ASD, ADHD, and schizophrenia

We also attempted to replicate previously reported associations with ASD, ADHD, and schizophrenia, testing all the alleles of specific genes based on previous implication of any allele of a given gene, rather than individual alleles. We chose this approach because many studies reported associations with serotypes or alleles using a two-digit resolution, rather than alleles/subtypes with a four-digit resolution, as used in this study. All allele-specific tests were subject to FDR correction, as described previously. For ASD and ADHD, we tested a subset of the genes (one or more of *HLA-A*, *HLA-B*, and *HLA-DRB1*), as previously reported associations between HLA genes and those disorders pertained mostly to those genes [5–8]. For schizophrenia, we tested the alleles of all genes, as associations with alleles of all of the HLA genes in our study have been reported in at least one study [10]. The full results of the replication analysis can be found in Supplementary Table S4. All alleles had *q* > 0.05 both in the gene-disorder groups and across all groups; similarly, no association survived Bonferroni correction. Nominally-significant associations (*p* ≤ 0.05) can be found in Table 4. None of the associations for ASD or ADHD in Table 4 correspond to the previously implicated alleles/serotypes, based on WHO grouping from the HLA Dictionary [27]. For schizophrenia, there is a nominally-significant positive association with DQB1*0604; a negative association with DQB1*0602 and schizophrenia in African Americans has been reported [28]. DRB1*0404 is negatively associated with schizophrenia in our study. This allele belongs to the serotype group DR4, which has been shown to be negatively associated with schizophrenia in two studies [29, 30]. Given the year of publication of the latest extensive review article, we used PubMed to search for schizophrenia studies that mentioned significant associations with the HLA alleles in Table 4 not mentioned by Wright et al. For A*0101, which belongs to serotype A1 and had a protective effect in our study, the previous literature is inconclusive as to its effects in schizophrenia, with some studies finding it increased risk and others finding it reduced risk [31].

**Table 4:** Nominally-significant associations (*p* ≤ 0.05) from the replication analysis; *p*-values and *q*-values in this table are rounded up to the third decimal place

Disorder	Allele	WHO-assigned	OR	*p*-value
ADHD	DRB1*1101	DR11	0.40	0.029
ASD	A*1101	A11	1.1	0.016
ASD	B*2702	B27	2.15	0.005
ASD	DRB1*0301	DR17	0.91	0.015
ASD	DRB1*1101	DR11	0.38	0.029
Schizophrenia	A*0101	A1	0.89	0.019
Schizophrenia	A*3402	A34	12.01	0.042
Schizophrenia	A*6801	A68	1.17	0.048
Schizophrenia	B*4701	B47	2.09	0.005
Schizophrenia	B*0801	B8	0.85	0.018
Schizophrenia	DQB1*0604	DQ6	1.18	0.013
Schizophrenia	DRB1*1104	DR11	1.57	0.034
Schizophrenia	DRB1*0404	DR4	0.39	0.046

## Discussion

Taken together, the results of the *HLA-DPB1* analyses in the various ASD and ID subgroups suggest that DPB1*1501 has a protective effect which can be identified mainly when comparing mentally-healthy controls with individuals with a severe phenotype consisting of a diagnosis of both ASD and ID, and it could be that it was mostly the latter group that was driving the previous associations with this allele. DPB1*1501 is a rare allele and it has not been extensively written about in the literature. A nominally significant association (not surviving correction for multiple testing) was reported for this allele in the autoimmune disease chronic idiopathic thrombocytopenia [32]. Even though the results of this study are not conclusive, it might be worth to mention the connection between autoimmunity and neurodevelopmental disorders such as ASD, which has been identified in both Danish and Swedish population studies [33, 34]. In another study, the protein product of *HLA-DPB1* has been shown to co-precipitate with a biologically-active form of the macrophage migration inhibitory factor (MIF) [35]. MIF acts as an inflammatory cytokine, and it has been implicated in inflammatory and autoimmune diseases [36]. Interestingly, a connection between MIF and autism has been reported, whereby higher levels of plasma MIF were correlated with autistic behavior [37]. While the functional mechanism behind the association of DPB1*1501 with reduced risk of a severe ASD-ID comorbidity is unclear, this and the above independent associations between *HLA-DPB1*, MIF, and neurodevelopmental disorders suggest a possible role for this gene in the etiology of ASD and ID. Additionally, a study of biomarkers for early detection of schizophrenia reported significantly altered levels of plasma MIF in a panel of blood-based biomarkers [38].

The association of increased risk with B*5701 was observed in all investigated ID groups except for the group in which all cases had a comorbid diagnosis of ASD. It seems that this association is driven by individuals who have ID but not ASD. This may suggest that B*5701 is a risk allele specific to a form of ID that is distinct from ASD and is not diagnosed together with it. However, there is also some association signal for this allele within ADHD cases and depression cases. Unlike the associations of *HLA-DPB1* with ASD and ID, the gene-based tests for HLA-B with ADHD or depression are not significant. Nonetheless, it is possible that the association with B*5701 in this sample is influenced by the presence of a diagnosis of ADHD or depression in addition to ID. Regarding any functional studies of this allele, it is known to be associated with hypersensitivity to an anti-retroviral drug [39]. B57 was noted in a study of specific language impairment (protective effect), although this association did not survive FDR correction [40]. We note again that, when a strict multiple testing correction (Bonferroni correction) was followed, only the association between DPB1*1501 and ID survived.

We did not find FDR-significant evidence for replication for previously reported associations between HLA alleles and ASD, ADHD, or schizophrenia. In the ADHD and ASD studies mentioned above, the sample sizes were small (fewer than 200 ASD cases in studies implicating *HLA-A* and *HLA-DRB1* alleles, and fewer than 400 cases in the study implicating *HLA-B*; for ADHD, the study implicating *HLA-DRB1* included fewer than 50 cases). In contrast, our sample sizes for ASD and ADHD are larger than 10,000 post-QC The only nominally-significant association that showed the same trend of association was with DRB1*0404, which belongs to the DR4 serotype, and schizophrenia, where the allele was protective. However, even this association, found by several studies, was not always replicated by others [41]. Thus, our results by large do not provide strong evidence of replication for most previously reported associations between ASD, ADHD, and schizophrenia and HLA genes. The lack of significant association between schizophrenia and HLA alleles in this large-scale study of a homogeneous population is not in line with many previous studies and requires further consideration. There could be several reasons for this discrepancy. Firstly, our sample size was much larger than in previous studies, and, with some studies having very small sample sizes, that could have led to false positives in those studies (the sample sizes in the studies that reported the association with DR4, for example, were quite small: fewer than 100 cases in one, and fewer than 300 cases in the other, compared to more than 2000 cases in our study). Differences in study design might also contribute to this effect: the statistical models used, the resolution of the HLA typing, the use of grouped alleles, based on serological activity, or individual alleles, and population-related differences could all potentially explain at least some of these differences. For example, a recent study that found an extremely significant association with schizophrenia used SNPs and not HLA types, and the index SNP that fell within the HLA region was not inside a known HLA gene, which may suggest a different mechanism underlying this association [42]. With regards to population differences, it should be mentioned that the HLA region varies greatly between different populations due to its evolutionary importance [43–45], which could also have an impact on association studies. Lastly, schizophrenia has been associated with excess occurrence of infections and autoimmune diseases [46], the latter of which also show association with HLA genes; however, this association might be due to environmental factors or other immune-related genes not captured by the HLA region itself; it has been previously shown that a polygenic risk score for schizophrenia does not predict risk of acquiring infections [47].

### Strengths and limitations

Our study cohort is population-based and genetically homogenous. It includes a larger sample than most immunogenetic studies of psychiatric and neurodevelopmental disorders, even for our smaller case groups, and diagnoses based on nation-wide criteria. As such, it offers a unique opportunity to study the role of the HLA genes in the etiologies of psychiatric and neurodevelopmental disorders. However, we note that for some of the diagnoses, namely the adult-onset ones, this sample might be relatively young. Our large sample sizes allowed us to examine rare alleles, but this, in turn, makes it hard to find suitable replication cohorts. We employed strict QC measures for both the imputation and association analyses, but we acknowledge that our results should be replicated in other studies. Lastly, it should be mentioned that in some cases alleles across more than one HLA gene may act in concert to increase or reduce risk. Given the approach employed in this study, we analyzed a large number of alleles and phenotypes and did not examine haplotypes across genes, but this can provide an interesting avenue of research in studies focusing on specific variants. We hope our study will prompt further investigations and make psychiatric cohorts in general and ID cohorts in particular more accessible. In conclusion, this study is, to our knowledge, the largest HLA association study for psychiatric and neurodevelopmental disorders. The results implicate HLA genes in the etiologies of ASD and ID. Furthermore, our study highlights the importance of large samples and consistent phenotypes in HLA association studies, as we did not obtain significant evidence of replication for previously-reported associations. Further studies will be required both to replicate the associations we have found and to elucidate the molecular mechanisms that underlie them.

## Supplementary information

Supplementary Figure S1

Supplementary Figure S2

Supplementary Figure S3

Supplementary Table S1

Supplementary Table S2

Supplementary Table S3

Supplementary Table S4
