# Correction: Immunity and mental illness: findings from a Danish population-based immunogenetic study of seven psychiatric and neurodevelopmental disorder

**DOI:** 10.1038/s41431-020-00772-y

**Published:** 2020-12-08

**Authors:** Ron Nudel, Michael E. Benros, Morten Dybdahl Krebs, Rosa Lundbye Allesøe, Camilla Koldbæk Lemvigh, Jonas Bybjerg-Grauholm, Anders D. Børglum, Mark J. Daly, Merete Nordentoft, Ole Mors, David M. Hougaard, Preben Bo Mortensen, Alfonso Buil, Thomas Werge, Simon Rasmussen, Wesley K. Thompson

**Affiliations:** 1 Institute of Biological Psychiatry, Mental Health Centre Sct. Hans, Mental Health Services Copenhagen, Roskilde, Denmark; 2 iPSYCH, The Lundbeck Foundation Initiative for Integrative Psychiatric Research, Copenhagen, Denmark; 3 Mental Health Centre Copenhagen, University of Copenhagen Hospital, Copenhagen, Denmark; 4 Department of Bio and Health Informatics, Technical University of Denmark, Kongens Lyngby, Denmark; 5 Center for Neonatal Screening, Department for Congenital Disorders, Statens Serum Institut, Copenhagen, Denmark; 6 Department of Biomedicine, Aarhus University and Centre for Integrative Sequencing, iSEQ, Aarhus, Denmark; 7 Aarhus Genome Center, Aarhus, Denmark; 8 Stanley Center for Psychiatric Research, Broad Institute of Harvard and MIT, Cambridge, MA USA; 9 Department of Clinical Medicine, Faculty of Health and Medical Sciences, University of Copenhagen, Copenhagen, Denmark; 10 Psychosis Research Unit, Aarhus University Hospital, Risskov, Denmark; 11 National Center for Register-Based Research, Aarhus University, Aarhus, Denmark; 12 Novo Nordisk Foundation Center for Protein Research, Faculty of Health and Medical Sciences, University of Copenhagen, Copenhagen, Denmark; 13 Department of Family Medicine and Public Health, Division of Biostatistics, University of California, San Diego, CA USA

Correction to: *European Journal of Human Genetics*

10.1038/s41431-019-0402-9

In the text of the original article linked to this correction article, we the authors include the ICD-10 codes for the diagnoses we used. We would also like to add the corresponding ICD-8 codes for some of the diagnoses, as a minority of the individuals might have received them while ICD-8 was being used in Denmark. This does not change the results or conclusions of the paper. However, for the sake of completeness, we believe the text should mention the codes that would have been used in those cases, in case the reader may need to look them up and/or compare them to studies which used ICD-8. Since the association with intellectual disability was the main new result from the paper, and some individuals received this diagnosis at a young age, unlike e.g. schizophrenia, they could have received an ICD-8 code (ICD-8 was used until 1994 and the first year of birth for the cohort is 1981), so it is important to note the codes used in those cases. We apologize for their omission from the original text. The original and revised text is as follows:

Original text (page 2 of the online PDF; page 1,446 of the printed PDF):

The phenotype groups included in this study were as follows: controls (no psychiatric or neurodevelopmental diagnoses, as per ICD-10 codes F00–F99); schizophrenia cases (F20); bipolar disorder (BPD) cases (F30–F31); single and recurrent depressive disorder cases (F32–F33); anorexia nervosa cases (F50.0); ASD cases (F84.0, F84.1, F84.5, F84.8, and F84.9), ADHD cases (F90.0) and intellectual disability (ID) cases (F70–F79).

Revised text:

The phenotype groups included in this study were as follows: controls (no psychiatric or neurodevelopmental diagnoses, as per ICD-10 codes F00–F99; equivalent ICD-8 codes 290–315); schizophrenia cases (F20; 295.x9 excluding 295.79); bipolar disorder (BPD) cases (F30–F31; 296.19, 296.39, 298.19); single and recurrent depressive disorder cases (F32–F33; 296.09, 296.29, 298.09, 300.49); anorexia nervosa cases (F50.0; 306.50); ASD cases (F84.0, F84.1, F84.5, F84.8, and F84.9), ADHD cases (F90.0) and intellectual disability (ID) cases (F70–F79; 311.xx, 312.xx, 313.xx, 314.xx, 315.xx).

