# Supplementary material for: Immunity and mental illness: findings from a Danish population-based immunogenetic study of seven psychiatric and neurodevelopmental disorders
Source: Eur J Hum Genet. 2019 Apr 11;27(9):1445–55. doi: 10.1038/s41431-019-0402-9 (PMC6777475; doi:10.1038/s41431-019-0402-9)
Supplement: Supplementary file 3 — Supplementary Figure S3 [file 41431_2019_402_MOESM3_ESM.pdf]

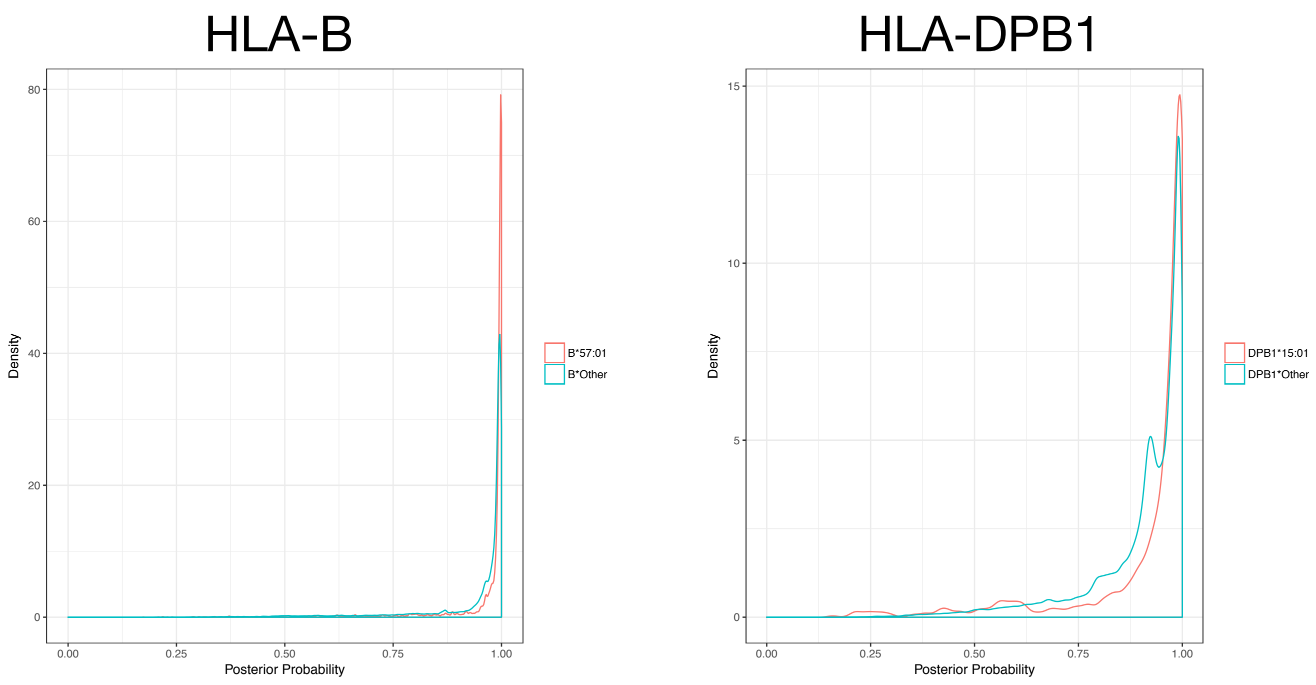

Supplementary Figure 3. HIBAG posterior probability density distributions for the significant alleles are shown in red (B*57:01 and DPB1*15:01). The other alleles from the respective locus are combined and shown in blue.
